# K-point longitudinal acoustic phonons are responsible for ultrafast intervalley scattering in monolayer MoSe_2_

**DOI:** 10.1038/s41467-022-32008-6

**Published:** 2022-07-25

**Authors:** Soungmin Bae, Kana Matsumoto, Hannes Raebiger, Ken-ichi Shudo, Yong-Hoon Kim, Ørjan Sele Handegård, Tadaaki Nagao, Masahiro Kitajima, Yuji Sakai, Xiang Zhang, Robert Vajtai, Pulickel Ajayan, Junichiro Kono, Jun Takeda, Ikufumi Katayama

**Affiliations:** 1 Laboratory for Materials and Structures, Tokyo Institute of Technology, Yokohama, Japan; 2 Department of Physics, Graduate School of Engineering Science, Yokohama National University, Yokohama, Japan; 3 School of Electrical Engineering, Korea Advanced Institute of Science and Technology (KAIST), Daejeon, Korea; 4 International Center for Materials Nanoarchitectonics (MANA), National Institute for Materials Science (NIMS), Tsukuba, Japan; 5 Department of Condensed Matter Physics, Graduate School of Science, Hokkaido University, Sapporo, Japan; 6 Institute of Laser Engineering, Osaka University, Osaka, Japan; 7 Department of Materials Science and NanoEngineering, Rice University, Houston, TX USA; 8 Department of Electrical and Computer Engineering, Rice University, Houston, TX USA; 9 Department of Physics and Astronomy, Rice University, Houston, TX USA

**Keywords:** Condensed-matter physics, Two-dimensional materials, Ultrafast photonics

## Abstract

In transition metal dichalcogenides, valley depolarization through intervalley carrier scattering by zone-edge phonons is often unavoidable. Although valley depolarization processes related to various acoustic phonons have been suggested, their optical verification is still vague due to nearly degenerate phonon frequencies on acoustic phonon branches at zone-edge momentums. Here we report an unambiguous phonon momentum determination of the longitudinal acoustic (LA) phonons at the K point, which are responsible for the ultrafast valley depolarization in monolayer MoSe_2_. Using sub-10-fs-resolution pump-probe spectroscopy, we observed coherent phonons signals at both even and odd-orders of zone-edge LA mode involved in intervalley carrier scattering process. Our phonon-symmetry analysis and first-principles calculations reveal that only the LA phonon at the K point, as opposed to the M point, can produce experimental odd-order LA phonon signals from its nonlinear optical modulation. This work will provide momentum-resolved descriptions of phonon-carrier intervalley scattering processes in valleytronic materials.

## Abstract (web summary)

Valley depolarization processes in 2D transition metal dichalcogenides have been linked to acoustic phonons, but optical verification is ambiguous, due to the nearly degenerate acoustic phonon frequencies at the zone-edge. Here, the authors determine the phonon momentum of the longitudinal acoustic (LA) phonons at the K point as responsible for the ultrafast valley depolarization in monolayer MoSe_2_.

## Introduction

**Fig. 1:**
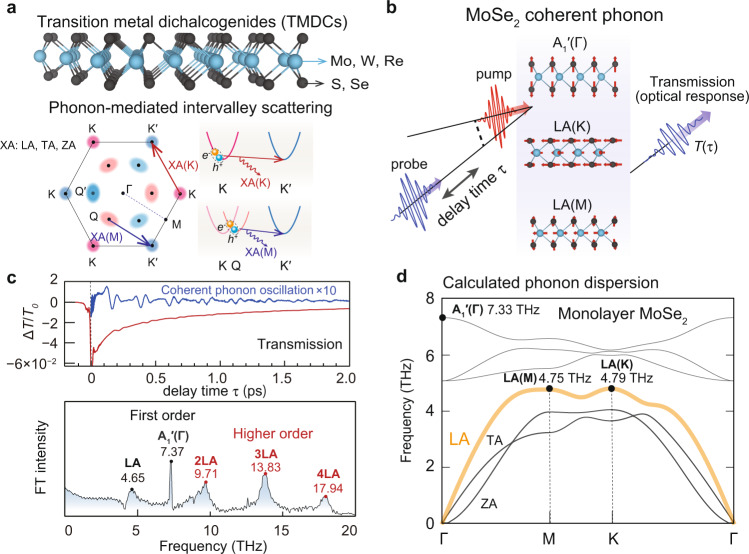
Schematic illustration of phonon-mediated intervalley scattering processes and experimental ultrafast pump-probe detection of monolayer MoSe_2_. **a** The atomic structure of transition metal dichalcogenides (TMDCs) and valley scattering processes mediated by XA(K) and XA(M) phonons (XA: LA, TA, and ZA). **b** Schematic illustration of the pump-probe detection of the ultrafast optical response of monolayer MoSe_2_ modulated by coherent phonon generation. **c** The experimental differential transmittance (upper panel) and its Fourier-transformed spectrum (lower panel) exhibiting coherent phonon signals in first-order A_1_′(Γ) mode and first- and higher orders of the LA mode. **d** The calculated phonon dispersion of monolayer MoSe_2_ obtained by density-functional theory. The A_1_′(Γ), LA(M), and LA(K) modes observed at 7.33, 4.75, and 4.79 THz are highlighted.

Phonon-mediated intervalley scattering is a central process in photoexcited carrier dynamics of valleytronic materials^1,2^. In transition metal dichalcogenides (TMDs), a prototypical family of valleytronic materials, it has been reported that the degree of valley polarization exhibits ultrafast decays in picosecond time scale due to intervalley carrier-phonon scattering^3–12^. In such scattering processes, zone-edge acoustic phonons play a definitive role in transferring photoexcited carriers from one valley to another; acoustic phonons at the K-point scatter carriers from K to K′ valleys (or K′ to K valleys) and those at the M point scatter carriers from Q to K′ valleys (or Q′ to K valleys) due to the momentum conservation (see Fig. 1a). While numerous optical experiments have been performed to probe zone-edge acoustic phonons by using resonant Raman scattering^13–18^, photoluminescence excitation^4,19^, and coherent phonon generation^20–22^, the phonon momentum have often remained largely unidentified because the frequencies of phonon modes at the K and M points TMDs are nearly degenerate (see Table 1). For instance, the LA phonon frequencies at the M and K points in Mo-based TMDs (MoS_2_ and MoSe_2_) and the ZA phonon frequencies at the M and K points of W-based TMDs (WS_2_ and WSe_2_) are nearly degenerated (frequency differences <0.1 THz = 0.4 meV), which are hardly resolved with the phonon frequencies obtained by typical linear spectroscopies.

**Table 1:** Phonon frequencies of acoustic branches (LA, ZA, and TA) at the M and K points in several transition metal dichalcogenides

	*ω*(M) (THz)	*ω*(K) (THz)	*ω*(K) − *ω*(M) (THz)
**MoSe**_**2**_			
LA	4.76	4.79	**0.04**
ZA	3.95	4.03	**0.08**
TA	3.21	3.63	0.42
**WSe**_**2**_			
LA	3.84	4.22	0.38
ZA	3.62	3.69	**0.06**
TA	2.91	2.93	**0.0**
**MoS**_**2**_			
LA	7.05	7.05	**0.00**
ZA	5.16	5.40	0.24
TA	4.67	5.51	0.85
**WS**_**2**_			
LA	5.32	5.52	0.20
ZA	4.28	4.29	**0.02**
TA	3.90	4.44	0.54

ω(M) and ω(K) denote the phonon frequencies at the M and K points and ω(K) − ω(M) are their frequency differences, in THz. Frequency differences lower than the frequency resolution of conventional femtosecond pump-probe measurements (typically, 0.1 THz = 0.4 meV) are marked in bold.

## Results

### Coherent phonon measurement

Here, we report on unambiguous determination of the phonon mode that dominates intervalley scattering in MoSe_2_: the K-point LA phonon. This conclusion was obtained through phonon-symmetry analysis and first-principles calculations, combined with nonlinear coherent phonon (CP) measurement, whose principle is schematically shown in Fig. 1b. The transmission modulations induced by CPs were monitored in the time domain, which was Fourier transformed to generate a CP spectrum (see Fig. 1c). On the basis of comparison with the calculated phonon dispersions of monolayer MoSe_2_ in Fig. 1d, we assign the observed CP peaks at 4.65 and 7.37 THz to the first-order LA mode and the optical A_1_′(Γ) mode, while the higher-frequency features (>10 THz) are attributed to multiple-order LA phonon modes: 2LA, 3LA, and 4LA. By contrast, the optical A_1_′(Γ) mode only shows a first-order CP signal (7.37 THz). This implies that the particular zone-edge LA phonon mode produced by ultrafast intervalley scattering possesses a characteristic nonlinear optical response, while the optical A_1_′(Γ) phonon only induces linear optical modulation. In what follows we show that this nonlinear optical response is the key feature that conclusively tells us that the generated LA phonons are at the K point, not at the M point.

### Phonon displacements and symmetry

**Fig. 2:**
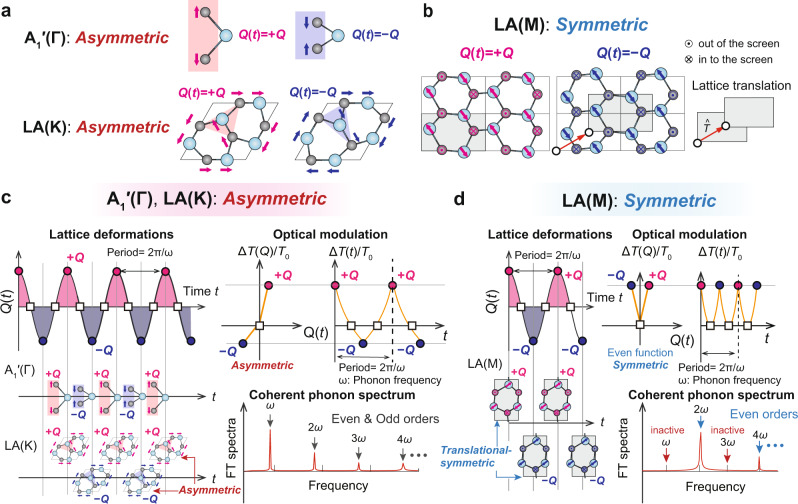
Lattice deformations of the A_1_′(Γ), LA(K), and LA(M) modes in TMDCs and their optical modulations (e.g., differential transmittance *ΔT/T*_0_) associated coherent phonon (CP) spectra. **a** *Asymmetric* lattice deformations of the A_1_′(Γ) and LA(K) modes. *Q*(*t*) represents the atomic displacements oscillating over time. The + and − signs of *Q*(*t*) denote the directions of vibrations. Lattice deformations of the A_1_′(Γ) and LA(K) modes are *asymmetric* because the atomic structures with +*Q* and −*Q* displacements are not identical. **b** *Symmetric* lattice deformations of the LA(M) mode. In contrast with the *asymmetric* A_1_′(Γ) and LA(K) modes, the lattice deformations of +*Q* and −*Q* displacements of the LA(M) mode superimposed after the lattice translation are identical. **c** Lattice oscillations of the *asymmetric* A_1_′(Γ) and LA(K) modes, with derived optical responses and CP spectra. As +*Q* and −*Q* displacements yield *asymmetric* atomic structures, nonlinear optical modulations with respect to the phonon frequency *ω* can be recorded as higher-order CP signals. **d** Lattice oscillation of the *symmetric* LA(M) mode, the derived optical response and CP spectrum exhibiting only even orders of the phonon frequency, due to the even function behavior of the optical response.

To clarify the nonlinear optical response and zone-edge LA phonon momentum intimately related to their optical processes, we first present a theoretical exploration of the lattice deformations and deformed atomic structures of the A_1_′(Γ), LA(K), and LA(M) phonon modes in monolayer MoSe_2_ using density-functional theory. Figure 2 shows lattice deformations and derived optical modulations of the A_1_′(Γ), LA(K) and LA(M) modes in monolayer MoSe_2_, with atomic displacements of the phonon modes calculated by density-functional theory (see Methods). As all monolayer TMDCs have the same type of atomic structure, the same description can be generally applied to other TMDCs. Figure 2a, b shows the lattice deformations and displaced atomic structures induced by the phonon modes, with *Q*(*t*) denoting the degree of atomic displacements at time *t*, which oscillates with the phonon frequency. We illustrate the displaced atomic structures of monolayer MoSe_2_ with lattice deformations of *Q*(*t*) = +*Q* and −*Q*, where + and − signs denote directions of the phonon vibrations. While all phonon modes deform the lattice structure from the equilibrium structure, we found a significant difference between lattice deformations in the LA(M) mode and both the A_1_′(Γ) and LA(K) modes. +*Q* and −*Q* displacements induced by the A_1_′(Γ) and LA(K) modes result in different atomic deformations, as shown in Fig. 2a, but those of the LA(M) mode can be superimposed after the lattice translation shown in Fig. 2b. Thus, +*Q* and −*Q* displacements induced by the A_1_′(Γ) and LA(K) modes are asymmetric, but those of the LA(M) modes always induce symmetric atomic structures.

The optical modulations induced by the coherent phonon generation of *asymmetric* and *symmetric* lattice deformations of the A_1_′(Γ), LA(K), and LA(M) modes and associated CP spectra are shown in Fig. 2c, d. Deformations of the atomic structure of monolayer MoSe_2_ by cosinusoidal lattice oscillations induced by coherent phonon vibrations are illustrated in the left-lower panels in Fig. 2c, d. As the asymmetric lattice deformations of the A_1_′(Γ) and LA(K) modes differ, the differential transmittance Δ*T*/*T*_0_ at the +*Q* and −*Q* displacements should have different values, as shown in Fig. 2c. The time evolution of *ΔT/T*_0_ is expected to oscillate with the phonon frequency *ω*. However, its shape is not a perfect cosinusoidal wave, leading to induction of the higher orders of *ω* such as 2*ω*, 3*ω*, 4*ω,* and so on, as obtained by Fourier transformation. By contrast, linear behavior of the differential transmittance *ΔT*(*Q*)/*T*_0_ can derive a perfect cosinusoidal optical modulation *ΔT*(*t*)/*T*_0_ ∝ *Q*(*t*) ∝ cos(*ωt* + *φ*) (where *φ* is a phase shift), yielding only first-order CP signals at the phonon frequency *ω*. This indicates that the nonlinear optical modulation of LA(K) modes is responsible for single and higher-order CP signals. On the other hand, because LA(M) phonons deform the atomic structure in the same crystallographic structure at both the +*Q* and −*Q* displacements, the differential transmittance *ΔT*(*Q*)/*T*_0_ should be an even function, i.e., Δ*T*( + *Q*)/*T*_0_ = Δ*T*(−*Q*)/*T*_0_, as illustrated in Fig. 2d. Suppose that the *ΔT*(*Q*)/*T*_0_ has been expanded with a polynomial of *Q*, i.e., Δ*T*(*Q*)/*T*_0_ = *a*_0_ + *a*_1_*Q* + *a*_2_*Q*^2^ + *a*_3_*Q*^3^ + *a*_4_*Q*^4^ …, the odd-order coefficients (for example *a*_1_ and *a*_3_) must be zero for the LA(M) mode due to the even function behavior of Δ*T*(*Q*)/*T*_0_. If an atomic displacement *Q*(*t*)∝sin(*ωt* + *φ*) is plugged into the Δ*T*(*Q*)/*T*_0_, the differential transmittance exhibits only even orders of the phonon frequency *ω* (see *Methods* for details). This indicates that the LA(M) mode is not responsible for the odd-order LA signals, our experimental CP spectrum is therefore attributable to dominant generation of LA(K) phonons, which are responsible for phonon-mediated ultrafast intervalley carrier scattering in monolayer MoSe_2_^7,20,23^. In turn, the striking difference between the LA(K) and A_1_′(Γ) modes observed in the CP spectrum is attributable to the linear and nonlinear behavior of the differential transmittance *ΔT*(*Q*)/*T*_0_. Seeking numerical details of the differential transmittance associated with the CP spectra, we next explicitly evaluate the differential transmittance induced by the A_1_′(Γ), LA(K) and LA(M) coherent phonons from the absorption spectra calculations using density-functional theory.

### Phonon-mediated optical modulation

**Fig. 3:**
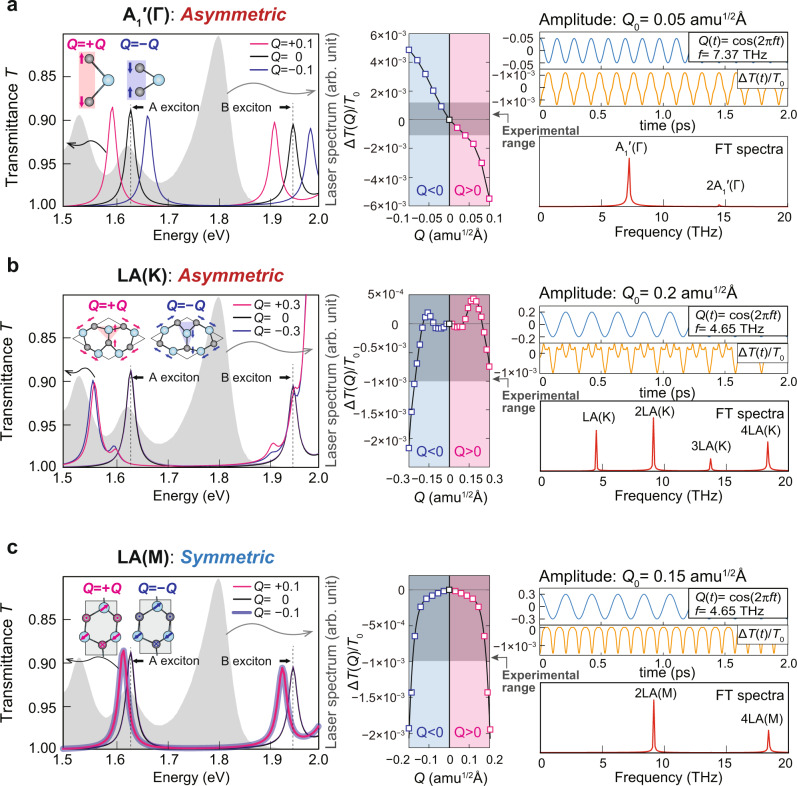
Calculated transmittance spectra of monolayer MoSe_2_ modulated by lattice deformations and derived optical modulations. The optical modulations are induced by: **a** A_1_′(Γ), **b** LA(K), and **c** LA(M) modes. Left panel: transmittance spectra of monolayer MoSe_2_ with and without the lattice deformations (*Q* = ± 0.1, ±0.3, and 0, respectively) and the laser spectrum (gray shading) used in the experiment. Middle panel: the differential transmittance *ΔT*(*Q*)/*T*_0_ as a function of the generalized displacement *Q* evaluated by integrating the experimental laser spectrum with the calculated transmittance spectra (see *Methods*). Right panel: simulated time-evolved differential transmittance *ΔT*(*t*)/*T*_0_ and associated CP spectra obtained by Fourier transformation of *ΔT*(*t*)/*T*_0_.

Figure 3 presents the transmittance spectra, the differential transmittance *ΔT*/*T*_0_ and simulated CP spectra of the A_1_′(Γ), LA(K), and LA(M) modes calculated by linear-response time-dependent density-functional theory (LR-TDDFT) using the HSE06 functional including spin-orbit coupling (SOC). We here quantify the degree of atomic displacements *Q* by introducing a generalized atomic displacement defined as *Q*^2^ = *Σm*_*i*_*d*_*i*_^2^ evaluated for the MoSe_2_ formular unit (three atoms), where *m*_*i*_ and *d*_*i*_ are the mass and displacement of the *i*-th atom in atomic mass units (amu) and angstroms (Å), respectively. The calculated transmittance with the atomic displacement of each phonon mode is shown in the left panels of Fig. 3. The A-exciton position of the calculated transmittance spectra is calibrated to the experimental value^20,24^. The asymmetric A_1_′(Γ) and LA(K) modes modulate each transmittance spectrum with +*Q* and −*Q* displacements shown in Fig. 3a, b (magenta and blue lines, respectively), while the transmittance spectra associated with +*Q* and −*Q* displacements of the LA(M) phonon are the same (Fig. 3c). These numerical results corroborate our findings derived from the asymmetric and symmetric behaviors of the A_1_′(Γ), LA(K), and LA(M) modes previously explained and illustrated in Fig. 2. Using the calculated transmittance spectra, the differential transmittance is obtained by the overlap integration of the experimental laser spectrum and calculated transmittance (see *Methods*). The calculated differential transmittance *ΔT*(*Q*)/*T*_0_ is shown in the middle panels of Fig. 3. Differences in behavior of *ΔT*(*Q*)/*T*_0_ among the three phonon modes can be clearly seen. The Δ*T*(*Q*)/*T*_0_ of the A_1_′(Γ) mode responds almost linearly with respect to the displacement *Q* (in Fig. 3a), whereas the LA(K) mode exhibits a highly nonlinear modulation on Δ*T*(*Q*)/*T*_0_ (in Fig. 3b). The LA(M) mode exhibits the symmetric Δ*T*(*Q*)/*T*_0_ due to an even function of the displacement *Q* (in Fig. 3c). We note that the A_1_′(Γ) mode reduces and increases the band gap of monolayer MoSe_2_ with +*Q* and −*Q* displacements, respectively, whereas both LA(K) and LA(M) modes with +*Q* and −*Q* displacements only reduce the band gap.

By comparing the calculated Δ*T*(*Q*)/*T*_0_ in Fig. 3 to the experimentally determined differential transmittance in Fig. 1c, the range of the displacement *Q* of each phonon can be approximately estimated. The gray shading in the Δ*T*(*Q*)/*T*_0_ spectra presented in middle panels of Fig. 3 show the experimental range of the differential transmittance, Δ*T*/*T*_0_, with maximum and minimum values of around 1 × 10^−3^. This corresponds to *Q* values of the A_1_′(Γ), LA(K) and LA(M) modes of 0.05, 0.2, and 0.15 amu^1/2^ Å, respectively. We set these maximum displacements, *Q*_0_, as the amplitudes of cosinusoidal lattice motions of the phonon modes, as shown in the right-upper panels in Fig. 3a–c. Evolutions with time of the differential transmission Δ*T*(*t*)/*T*_0_ are then readily obtained from the calculated Δ*T*(*Q*)/*T*_0_ by inserting Q(*t*) = *Q*_0_cos*ωt* as the time-dependent displacement *Q*. Simulated CP spectra of the A_1_′(Γ), LA(K) and LA(M) modes are obtained from Fourier transformations of Δ*T*(*t*)/*T*_0_, as shown in the right-bottom panels of Fig. 3a–c. The simulated CP spectra explain the experimental CP spectra well, as follows. The first-order peak dominates the CP spectrum of the A_1_′(Γ) mode because its atomic motion monotonically increases and decreases the band gap of monolayer MoSe_2_. The higher-order CP signals of LA phonons originate from nonlinear behavior of the optical modulation induced by LA(K) phonons. Activation of the LA(M) mode in the CP spectrum is strictly limited to its even orders due to the symmetric lattice deformations of the +*Q* and −*Q* displacements. Thus, our numerical simulation resolves the acoustic momentum of LA phonons through the nonlinear and higher-order optical responses of monolayer MoSe_2_ induced by the LA(K) mode.

**Fig. 4:**
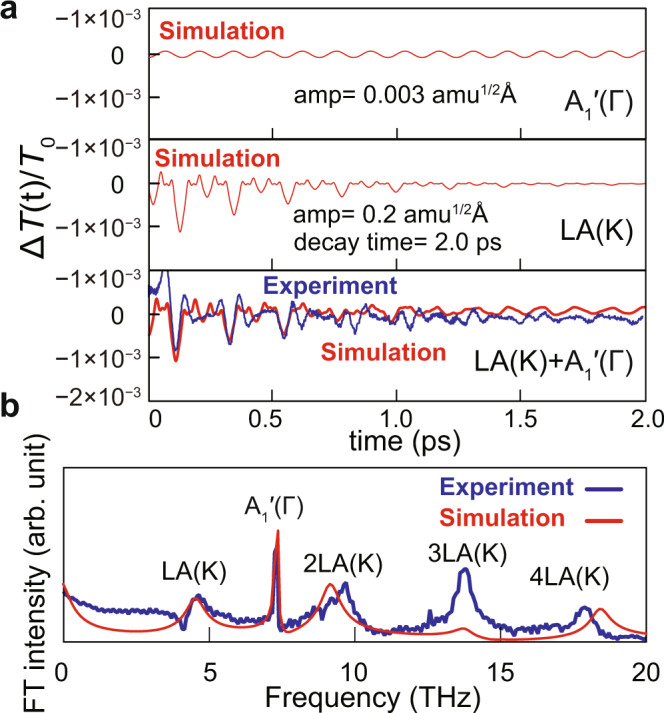
Simulated and experimental differential transmittance Δ*T*(*t*)/*T*_0_ and CP spectra. **a** Simulated differential transmittance Δ*T*(*t*)/*T*_0_ of the A_1_′(Γ) mode (upper panel), LA(K) mode (middle panel) and combination of the LA(K) and A_1_′(Γ) modes (bottom panel). Simulation parameters are shown in the insets. **b** Comparison of experimental and simulated CP spectra. The latter was obtained from the Fourier transformation of the differential transmittance Δ*T*(*t*)/*T*_0_ of the combination of LA(K) and A_1_′(Γ) modes (bottom panel of **a**).

We present a quantitative comparison of the experimental and simulated spectra in Fig. 4. To include the fast decay of the experimental Δ*T*(*t*)/*T*_0_, the single exponential decay of 2 ps has been imposed on the simulated Δ*T*(*t*)/*T*_0_ modulated by the LA(K) mode. The simulated Δ*T*(*t*)/*T*_0_ spectrum obtained with the combination of the LA(K) and A_1_′(Γ) modes quantitively reproduces the experimental *ΔT*(*t*)/*T*_0_ spectrum, as shown in the bottom panel of Fig. 4a. Here, amplitudes *Q*_0_ of the A_1_′(Γ) and LA(K) modes are set to 0.003 and 0.2 amu^1/2^ Å, whose atomic displacements of the A_1_′(Γ) and LA(K) modes are presented in Table 2. The large atomic displacements of the LA(K) mode compared to that of the A_1_′(Γ) mode indicates that intervalley scattering process would dominantly occur compared to the impulsive stimulated Raman scattering (ISRS)^21,23^. We showcase the simulated Fourier-transformed CP spectrum compared to the experimental CP spectrum in Fig. 4b, which reproduces overall characteristics of the experimental CP spectrum. The simulated spectrum comes from a combination of A_1_′(Γ) mode and LA(K) mode having a rapid damping of 2 ps, which could result in asymmetric shapes of Fourier signals. Some marginal inconsistencies remain e.g., relative intensity of the 3LA(K) mode and small variation of the 2LA(K) and 4LA(K) frequencies, which might be attributed to transient phonon frequency chirping through light-induced lattice strain.

**Table 2:** Maximum displacements of Mo and Se atoms in monolayer MoSe_2_ deformed by the A_1_′(Γ), LA(K), and LA(M) modes

	A_1_′(Γ) (*Q* = 0.003)	LA(K) (*Q* = 0.2)	LA(M) (*Q* = 0.15)
Mo	0.0000	0.0281	0.0187
Se	0.0007	0.0424	0.0189

The maximum differential transmittance induced by phonon modes was set to *ΔT*/*T*_0_ ≈ 1 × 10^−3^. The atomic displacements (in Å) and corresponding generalized atomic displacements are shown in parentheses (in amu^1/2^Å).

## Discussion

Finally, we briefly discuss the generation mechanism of the LA(K) phonon in monolayer MoSe_2_ observed in our experiment. It has been previously reported that K-point phonon generations in TMDs can involve multiple acoustic phonon branches. For instance, the LA(K) phonon has been widely detected in several TMDs^10,11,25^. In contrast, the coherent ZA(K) phonon generation has been reported for monolayer MoSe_2_ when the spin-flip intervalley scattering occurs in between lowest energy K-point valleys with opposite spins, resulting in the generation of the flexure (out-of-plane) ZA(K) phonons^20^. Because our laser spectrum (cf. gray shadings in Fig. 3) spreads over a wide excitation energy range which includes high energy excitations over the lowest energy valleys, intervalley carrier-phonon scatterings are not limited to the spin-flip intervalley scattering as in Ref. 20 but includes spin-conserved intervalley scatterings. The predominant occurrence of spin-conserved intervalley scatterings over the spin-flip intervalley scatterings then leads to the generation of the in-plane LA(K) phonons in our experiment rather than the flexural ZA(K) phonons as in Ref. 20. This demonstrate that, since the generation mechanism of zone-corner acoustic phonons is intimately related to the carrier excitation energy and spin relaxation, the nonlinear optical response should present an essential clue for exploring the valley depolarization process of TMDs.

In summary, we demonstrate higher-order optical responses of coherent phonon generation in monolayer MoSe_2_ that identifies the momentum of the LA phonon. The symmetric analysis of lattice deformations of coherent phonons decodes the higher-order optical response of zone-edge acoustic coherent phonons, and the higher-order coherent LA signals can be attributed to LA(K) phonons. Our first-principles calculations enable quantitative analysis of coherent phonon generation of the A_1_′(Γ) and LA(K) modes, revealing that acoustic LA(K) phonon generation via intervalley scattering dominates over generation of the A_1_′(Γ) mode through ISRS processes. Our work unveils hidden physics of the higher-order optical responses of monolayer MoSe_2_, thus facilitating deterministic descriptions of ultrafast phonon-mediated carrier scattering processes in a wide range of valleytronic materials.

## Methods

### Experimental setup and sample

Coherent phonon experiments with a degenerate pump-probe configuration were performed using a sub-10-fs Ti:sapphire laser (VENTEON PulseONE) with 7.5 fs pulse duration, 90 MHz repetition rate and 300 mW output power. The spectrum of the laser ranges from 650 nm (1.91 eV) to 1050 nm (1.18 eV) (see gray shadings in Fig. 3). The laser output was divided into pump (100 mW) and probe (5 mW) beams, both of which were simultaneously focused on the sample at approximately a normal incidence using a parabolic mirror with 50 mm focal length. The diameter of the focus at the sample was 20 μm. An optical shaker with a 15 ps scanning range running at 20 Hz was placed in the pump beam path, and changes in transmitted probe pulses were detected using a Si photodiode. After subtraction of the intensity of the reference probe pulses, the signal was amplified with a SR560 transimpedance current amplifier. The amplified signal was collected with an analog-to-digital converter and analyzed with the position signal sent from the optical shaker. All the measurements were performed at room temperature.

The sample consisted of monolayer MoSe_2_ crystals grown by the chemical vapor deposition technique on a sapphire substrate. The triangular single crystals of monolayer MoSe_2_ with the typical size of 200 μm were confirmed by optical microscopy. We implemented the in-situ microscope at the sample position of the setup for coherent phonon experiment and confirmed that the pump and probe beams are shined on the monolayer region of the sample. The reported waveforms were repeatedly observed during the experiments, indicating that no degradation of the sample occurred during the experiments. The absorption peak of the A-exciton resonance was observed around 1.6 eV in our sample, which was confirmed by a conventional transmission spectrometer.

### First-principles calculations

We calculated electronic and vibrational properties of monolayer MoSe_2_ using density-functional theory (DFT) with the projector augmented wave (PAW) method, implemented in the ﻿Vienna ab initio simulation package (VASP)^26–29^. The kinetic energy cut-off of plane waves was set to 350 eV and a Γ-centered 12 × 12 × 1 Monkhorst-Pack **k**-point grid was used for the primitive cell. For supercell calculations, Γ-centered 2 × 2 × 1 and 6 × 6 × 1 Monkhorst-Pack **k**-point grids were employed for 6 × 6 × 1 and $\sqrt 3\,\times\, \sqrt 3\,\times\, 1$ supercells, respectively. The phonon dispersion of monolayer MoSe_2_ and atomic displacements of the A_1_′(Γ), LA(K), and LA(M) modes were calculated using the Phonopy package^30^. Force constants used in the phonon dispersion calculation were generated with DFT calculations of displaced 6 × 6 × 1 supercells of the monolayer MoSe_2_ primitive cell using the PBEsol functional^31^. Optical properties of monolayer MoSe_2_ with and without atomic displacements of the phonon modes were obtained from the optical calculations of linear-response time-dependent density-functional theory (LR-TDDFT) with the HSE06 kernel including the spin-orbit coupling (SOC) effect as implemented in the VASP package^32^. Transmittance spectra of monolayer MoSe_2_ were extracted using VASPKIT code from the real and imaginary dielectric functions of the LR-TDDFT results^33^.

### Simulation of optical modulation and coherent phonon spectra

Integrated transmittance with a generalized atomic displacements *Q* are evaluated for monolayer MoSe_2_ as $T(Q)\,=\,\int {\widetilde{T}}_{Q}(\epsilon )I(\epsilon )d\epsilon$, where *ϵ* is the excitation energy, ${\widetilde{T}}_{Q}(\epsilon )$ is the calculated transmittance spectra of monolayer MoSe_2_ using LR-TDDFT with generalized atomic displacement *Q*, and *I*(*ϵ*) is the experimental lager spectrum used in the pump-probe experiment. The generalized atomic displacements are estimated as *Q*^2^ = Σ*m*_*i*_*d*_*i*_^2^, where *m*_i_ and *d*_i_ are the mass and displacement of the *i*-th atom in atomic mass units (amu) and angstroms (Å), respectively. A constant 0.1 eV downshift was applied to the excitation energy of the calculated transmittance ${\widetilde{T}}_{Q}(\epsilon )$ to adjust the A-exciton energy to the experimental value. The differential transmittance *ΔT*(*Q*)*/T*_*0*_ was calculated for various *Q* values with atomic displacements of A_1_′(Γ), LA(K), and LA(M) modes as *ΔT(Q)* = [*T*(*Q*) *−* *T*_*0*_]*/T*_*0*_, where *ΔT*(*Q*) and *T*_*0*_ are integrated transmittance spectra with generalized atomic displacements *Q* and *Q* = 0. The time-dependent differential transmittance *ΔT*(*t*)*/T*_*0*_ was obtained by giving a cosinusoidal oscillation *Q*(*t*) = *Q*_0_cos*ωt* into the *ΔT*(*Q*)*/T*_*0*_. For actual calculations of *ΔT*(*t*)*/T*_*0*_, the differential transmittance *ΔT*(*Q*)*/T*_*0*_ of each phonon mode was expanded into a polynomial of *Q* up to 6th order by numerical fitting. Finally, a simulated coherent phonon spectrum of each phonon mode was obtained by Fourier transformation of *ΔT*(*t*)*/T*_*0*_.

### Odd-order regulation of coherent phonon signals of the LA(M) mode

The differential transmission *ΔT*(*Q*)*/T*_*0*_ of the LA(M) mode is an even function which satisfies *ΔT*(+*Q*)*/T*_*0*_ = *ΔT*(−*Q*)*/T*_*0*_. Accordingly, the polynomial expansion of *ΔT*(*Q*)*/T*_*0*_ with respect to Q can be written as ΔT(*Q*)/T_0_ = *a*_0_ + *a*_2_*Q*^2^ + +*a*_4_*Q*^4^… (without odd orders of Q) where *a*_n_ is the *n*th-order coefficient. By giving *Q*(*t*) = *Q*_0_cos*ωt* to *ΔT*(*Q*)*/T*_*0*_, the time-dependent differential transmission is *ΔT*(*t*)*/T*_*0*_ =  *a*_0_ + *a*_2_cos^2^*ωt* + *a*_4_cos^4^*ωt*…. By adopting double angle identities of the cosine function cos^2^(*ωt*) = [1 + cos2(*ωt*)]*/*2 to cos^n^(*ωt*) terms, *ΔT*(*t*)*/T*_*0*_ can be reformulated into *ΔT*(*t*)*/T*_*0*_ = *b*_0_ + *b*_2_cos(2*ω*)*t* + *b*_4_cos(4*ω*)*t*…(where *b*_n_ is the *n*th coefficient) whose Fourier transformation coefficients [the coherent phonon spectrum of the LA(M) mode] only have even orders of the phonon frequency *ω*, such as 2*ω*, 4*ω*, 6*ω*… excluding odd orders.

## Data Availability

All relevant data are available from the authors upon reasonable request.
